# Antioxidant, Physicochemical and Rheological Properties of White and Milk Chocolate Compounds Supplemented with Plant-Based Functional Ingredients

**DOI:** 10.3390/foods13223694

**Published:** 2024-11-20

**Authors:** Elinda Okstaviyani, Puput Dwi Lestari, Kawiji Kawiji, Raden Baskara Katri Anandito, Anastriyani Yulviatun, Ardiba Rakhmi Sefrienda, Dimas Rahadian Aji Muhammad

**Affiliations:** 1 Department of Food Science and Technology, Faculty of Agriculture, Universitas Sebelas Maret, Jl. Ir. Sutami 36A Kentingan, Surakarta 57126, Indonesia; 2 Department of Agricultural Product Technology, Vocational School, Universitas Sebelas Maret, Jl. Kolonel Sutarto 150K, Jebres, Surakarta 57126, Indonesia; 3 Research and Development Center for Food, Nutrition and Public Health, Universitas Sebelas Maret, Jl. Ir Sutami 36A, Surakarta 57126, Indonesia; 4 Research Center for Food Technology and Processing, National Research and Innovation Agency (BRIN), Jl. Yogya Wonosari, Km. 31.5, Gunungkidul, Yogyakarta 55861, Indonesia

**Keywords:** antioxidant, butterfly pea flower, chocolate, sappan wood

## Abstract

Product development must be continuously done by the chocolate industry to face a high level of competitiveness in the market industry. This study investigates the effect of powdered sappan wood and butterfly pea flower incorporation in milk and white chocolate compounds. Four concentrations of each additional ingredient were used (0, 5, 10 and 15%). The results show that incorporating powdered sappan wood and butterfly pea flower significantly improved the total phenolic and flavonoid content and antioxidant activity of milk and white compounds. This study clearly shows that the selected plant could be an alternative to improve the health-promoting properties of milk and white chocolate compounds. However, supplementation also has some drawbacks, particularly in increasing the moisture content and the degree of colour difference between the milk and white compounds containing additional ingredients and the control. Also, powdered sappan wood and butterfly pea flower caused a higher viscosity of milk and white chocolate compounds. The results obtained in this study create a new strategy for using sappan wood and butterfly pea flower in various food products.

## 1. Introduction

Chocolate is a famous confectionery product around the globe, as it is strongly associated with a good mood, in addition to its pleasant sensory characteristics. It was reported that the annual consumption of chocolate is 8.13 million tonnes, with a market size of about USD 113.6 billion, and is expected to still grow at 3.7% by 2030 [1,2]. Due to its market potential, the chocolate industry has grown, resulting in a high level of competitiveness [3]. The chocolate industries, therefore, have endeavoured to add more value to the product to attract consumers. Making single-origin chocolate and replacing the milk content in chocolate by using plant-based material are two new trends in the chocolate industries [4,5]. Also, formula modifications of chocolate by supplementing various spices, probiotics and other functional ingredients have been reported in the literature [6,7,8]. The incorporation of spices is not only done in chocolate bars but also chocolate spreads and chocolate drinks [9,10].

Adding spices and some other functional materials to the chocolate formula aims to improve the chocolate’s health-promoting properties. This is based on the fact that cocoa, the main ingredient in chocolate production, is rich in bioactive compounds that benefit health [11]. However, the compounds significantly decrease during processing. Dark chocolate with a high cocoa content still retains substantial amounts of polyphenols. However, milk and white chocolates contain lower cocoa contents, making these products contain lower bioactive compounds than dark chocolate. White chocolate, made from cocoa butter, sugar, milk and emulsifier, contains almost zero phenolic content [12].

Sappan wood (*Caesalpinia sappan* L.) and butterfly pea flower (*Clitoria ternatea* L.) are two plant materials potentially used to improve chocolate’s health-promoting properties. Sappan wood is a plant of Leguminosae. It contains various bioactive compounds, one of which is brazilin, a natural red flavonoid [13]. Other important compounds in sappan wood include brazilein, sappan chalcone and protosappanin [14]. It has been reported that sappan wood has antioxidant, antibacterial, anti-inflammatory, hypoglycaemic and hepatoprotective properties. Also, sappan wood’s bioactive compounds may play a significant role in lowering inflammation, enhancing blood circulation, as well as reducing gastrointestinal problems and respiratory infection [14]. According to [15], sappan wood has been used for various purposes for centuries in numerous regions; even with the consumption level up to 5000 mg/kg, no side effects or fatalities have been documented. Furthermore, this study also mentioned that no animal experienced weight loss or organ failure during or after the 28-day experiment.

Meanwhile, butterfly pea flower, also well known as blue pea flower or Asian pigeon wings flower, is a family of Fabaceae in which the most used part is the flower [16]. Traditionally, the blue pigment of the flower is used as a colouring agent. Nowadays, the butterfly pea flower has been acknowledged to have antioxidant, antibacterial, antifungal activity, anti-inflammatory, antiproliferative, anticancer and antidiabetic activities [17]. As explained in this study, butterfly pea flower contains important minerals, such as calcium, magnesium, potassium, zinc, sodium and iron. Several bioactive compounds, such as alkaloids, tannins, glycosides, resins, steroids, saponins, flavonoids and phenols, mainly anthocyanins and various flavanol glycosides of kaempferol, rutin, quercetin and myricetin, have been previously identified in the flower by some research groups [18,19,20]. A long history of butterfly pea flower as food and beverage ingredients in various countries make this flower accepted as a food additive in ordinary foods in various regions, including Japan, Thailand and Taipei. Scientific evidence of the butterfly pea flower safety has been reported by [21], in which an ethanolic extract of the butterfly pea flower at a consumption level of 2000 mg/kg body weight showed no evidence of mortality or abnormalities without alteration of the haematological results.

Thus, supplementing sappan wood and butterfly pea flower in a chocolate formula seems a promising way to improve the health-promoting properties of the chocolate, including its phenols and antioxidant activity. The existing literature shows that scientists have conducted some attempts to enrich chocolates with various functional ingredients, including ω-3 PUFAs, probiotics, phenolic extracts, vitamins and minerals [22]. The supplementation of sappan wood and butterfly pea flower in a chocolate formulation has been carried out previously in our preliminary studies [23,24]. It was found that the supplementation of sappan wood and butterfly pea flower in a chocolate formula of up to 15% was acceptable in terms of sensory characteristics, even though it altered the hardness of the chocolates. The previous studies on the enrichment of sappan wood and butterfly pea flower in chocolate have not investigated the antioxidant activity of the chocolates. Also, the impact of sappan wood and butterfly pea flower on the other important quality attributes of chocolates has not been discussed. Many studies have reported that supplementing additional ingredients in a chocolate formula altered the characteristics of the chocolate, including its moisture content, appearance and rheological properties [11,25]. As such, a previous study has showed that white chocolate formulated with a microcapsule containing EPA and DHA caused colour alterations, in terms of brightness, hardness and whiteness index [7]. Another research showed that the incorporation of plant-based ingredients, such as Sakura green tea leaves and turmeric powder, significantly improved the total phenols and antioxidant activity of dark chocolate [26]. Meanwhile, adding encapsulated green tea extract in white chocolate has been reported to increase the viscosity of the chocolate [27]. Also, the addition of plant-based ingredients has been reported to increase the moisture content of chocolate [28]. Based on these backgrounds, therefore, this study aims to investigate the effect of sappan wood and butterfly pea flower supplementation in white and milk compound chocolate regarding the antioxidant properties, physicochemical properties, including moisture content and colour, and rheological properties, which are essential quality attributes in chocolate.

## 2. Materials and Methods

### 2.1. Preparation of Chocolate Samples

Milk compound chocolate and white compound chocolate from a brand of Tulip produced by PT Freyabadi Indotama (Indonesia) were used separately in this study. Powdered butterfly pea flower was obtained from Kusuka Ubiku (Bantul, Indonesia), while powdered sappan wood was obtained from Asyifa Herbal Sehat (Surakarta, Indonesia). All the materials are commercially available on the market.

The production of milk and white compound chocolate enriched with powdered butterfly pea flower and powdered sappan wood followed the previous published report with some modifications [11]. First, each chocolate type (800 g) was melted using steaming, called the Au Bain Marie method at a temperature of 60 °C. Afterwards, the melted chocolate was then mixed with powdered butterfly pea flower or powdered sappan wood with an 80-mesh particle size at a level of 0, 5, 10 and 15% (*w*/*w*) in a ECGC 12SLTA melanger (Cocoa Town, Alpharetta, GA, USA) and continued by using the melanging process for 1 h to reduce the particle size of the samples. After that, consecutive steps of moulding, cooling, demoulding and packing using aluminium foil were conducted. The tempering process was not conducted, because this research used compound chocolate that does not need a tempering process for solidification. Three independent samples were prepared for each concentration.

### 2.2. Extraction of Butterfly Pea Flower, Sappan Wood, Milk and White Compounds

The following procedure was used to extract the phenols and flavonoids from the samples before the analysis of the antioxidant properties. For powdered butterfly pea flower and sappan wood, the extraction was carried out using methanol (70%). For the milk and white compounds, the extraction method followed the technique used in the previous study [29]. An accurately weighted sample (5 g) was defatted using 100 mL of n-hexane thrice. The defatted sample was then air-dried in a dark condition for 24 h to remove the residues of n-hexane. An extractive solvent containing acetone (70%), distilled water (29.8%) and acetic acid (0.2%) was prepared. The defatted sample was extracted using the solvent at a ratio of 10:25 (*w*/*v*) for 30 min in an ultrasonic bath (Baku BK-2000, Baku, Guangzhou, China) at 40 kHz and 120 W set at room temperature, followed by centrifugation for 10 min at 3000 rpm using a Kokusan H-107 centrifuge (Kokusan Corp., Saitama, Japan) set at ambient temperature. This procedure was carried out twice, and the supernatant was collected and filtrated to remove the residual particles using Whatman paper #1.

### 2.3. Antioxidant Properties Analysis

#### 2.3.1. Analysis of the Total Phenolic Content

The extract obtained in the extraction step (200 µL) without any dilution was added to 200 µL Folin–Ciocalteu reagent previously mixed with 1 mL distilled water. After incubation for about 6 min, the mixture was added with 7% Na_2_CO_3_ solution (2.5 mL) and distilled water to reach the total volume of 6 mL. After incubation for 90 min at ambient temperature (20 °C) and dark conditions, the absorbance was measured at 760 nm in a UV–Visible spectrophotometer (Shimadzu BioSpec-1600, Shimadzu Corp., Kyoto, Japan). The total phenolic content was expressed as milligrams of gallic acid equivalent per gram sample (mg GAE/g sample) [11].

#### 2.3.2. Analysis of the Total Flavonoid Content

The total flavonoid content of the sample extracts was estimated to be the same as that of the previous study [30]. Shortly, aluminium chloride (5 mL, 0.1 M) was well mixed with the pure extract (200 µL). Afterwards, incubation for 40 min at room temperature was carried out, and then, the absorbance was determined at 415 nm. The total flavonoid content was expressed as milligrams of quercetin equivalent per gram of sample (mg QE/g sample).

#### 2.3.3. Analysis of DPPH (2,2-Diphenyl-1-picrylhydrazyl) Radical Scavenging Activity

The sample extract (300 µL) at a concentration of 10% was mixed with 4 mL of DPPH solution (0.3 mM). An incubation for 30 min was then carried out. The absorbance was then measured at 517 nm using a UV–Vis spectrophotometer. A similar treatment was conducted for the blank solution to obtain absorbance of the control. The DPPH radical scavenging activity was calculated using Equation (1), where Ac is the absorbance of the control, and As is the absorbance of the sample.

(1) $$\%inhibiton=\frac{\left(Ac-As\right)\times 100}{Ac}$$

#### 2.3.4. Analysis of Ferric Reducing Antioxidant Power (FRAP) Activity

A phosphate buffer (0.2 M, pH 7) was first prepared, and then, 2.5 mL of the buffer was mixed with 1 mL of the sample extract, followed by adding 1% potassium ferricyanide (2.5 mL). The mixture was then maintained in a water bath at 50 °C for 30 min. Afterwards, 2.5 mL of 10% trichloroacetic acid was added. Centrifugation for 10 min at 6500 rpm was done at room temperature to separate aggregates. The supernatant was mixed with distilled water and 0.1% of FeCl_3_ in a ratio of 5:5:1. The absorbance was measured at 700 nm in a UV–Visible spectrophotometer. The total phenolic content was expressed as milligrams of ascorbic acid equivalent per gram sample (mg AAE/g sample) [11].

### 2.4. Physicochemical Analysis

#### 2.4.1. Analysis of the Moisture Content

The moisture content analysis of all the samples was performed using the thermogravimetry method following the standard procedure of AOAC [31].

#### 2.4.2. Analysis of Colour

The samples’ colour analysis was measured using a Konica Minolta CR-400 Chroma Meter. The obtained values of lightness (L), a* and b* were then used to calculate the chroma (C*), °Hue, Whiteness Index (WI) and the degree of difference between the milk and white compounds containing additional ingredients and the control (∆E) using Equations (2)–(5), respectively, as also used in the previous study [11].

(2) $$C*=\sqrt{a^{*2}+b^{*2}}$$

°Hue = tan^−1^ (b*/a*) (3)

(4) $$WI=100-\sqrt{{\left(100-L*\right)}^{2}{+a*}^{2}{+b*}^{2}}$$

(5) $$∆E=\sqrt{{{{\left(L_{1}^{*}-L_{2}^{*}\right)}^{2}+(a_{1}^{*}-a_{2}^{*})}^{2}+(b_{1}^{*}-b_{2}^{*})}^{2}}$$

### 2.5. Rheological Properties Analysis

The rheology of the milk and white compounds was determined according to the ICA46 protocol, also used in our previous study [11]. The analysis used a Rotational Rheometer (RheolabQC, Anton Paar, Graz, Austria) coupled with a CC27 concentric cylinder system. The shear stress, apparent viscosity and torque were detected at the shear rate of 50 s^−1^, illustrating the viscosity of chocolate in the mouth during consumption.

### 2.6. Experimental Design and Statistical Analysis

The parameters that were considered to be affecting the characteristics of the chocolates were the concentrations of powdered butterfly pea flower or powdered sappan wood. The experiment was performed by means of a completely randomised design (CRD), and the results represented the means of three replicates. Statistical analysis was performed using IBM SPSS Statistics V22.0 (SPSS Inc., Chicago, IL, USA). One-way ANOVA was continued by Duncan’s Multiple Range Test (DMRT) to determine the effect of powdered butterfly pea flower or powdered sappan wood on each type of chocolate, with a confidence level of 95%.

## 3. Results and Discussion

### 3.1. Moisture Content and Antioxidant Properties of Sappan Wood and Butterfly Pea Flower

This research continues our previous studies. Based on the consumer acceptance evaluation in our previous studies, adding sappan wood powder at moderate concentrations was acceptable to consumers. However, high concentrations negatively affected the flavour and overall palatability [24]. This phenomenon was also found in the chocolate enriched with powdered butterfly pea flower. Moderate concentrations of the flower powder were well accepted in sensory evaluations [23]. The results offer the prospect of using sappan wood and butterfly pea flower in the chocolate industry. However, the impact of the concentration on other quality parameters of chocolates, such as antioxidants and rheology, was still unclear, and these were then investigated in this study. Our knowledge of the characteristics of raw materials is substantial enough to give a deeper insight into the quality of the final product. The moisture content and antioxidant properties of sappan wood and butterfly pea flower are shown in Table 1.

It was shown that powdered sappan wood and butterfly pea flower have a moisture content of 8.8% and 14.9%, respectively. These raw materials contained phenols and flavonoids, and thus, the materials exhibited antioxidant activity. The total phenolic and flavonoid content of sappan wood was higher than that of butterfly pea flower. Thus, the FRAP activity of sappan wood was higher than that of butterfly pea flower. Interestingly, in the DPPH radical scavenging activity, the butterfly pea flower was higher than sappan wood. This phenomenon may be because non-phenol compounds of butterfly pea flower contribute to DPPH radical scavenging activity. Anthocyanin is one of the main bioactive compounds of butterfly pea flower, demonstrating DPPH radical scavenging activity [32].

### 3.2. Antioxidant Properties of Chocolates Formulated with Sappan Wood and Butterfly Pea Flower

**Figure 1:**
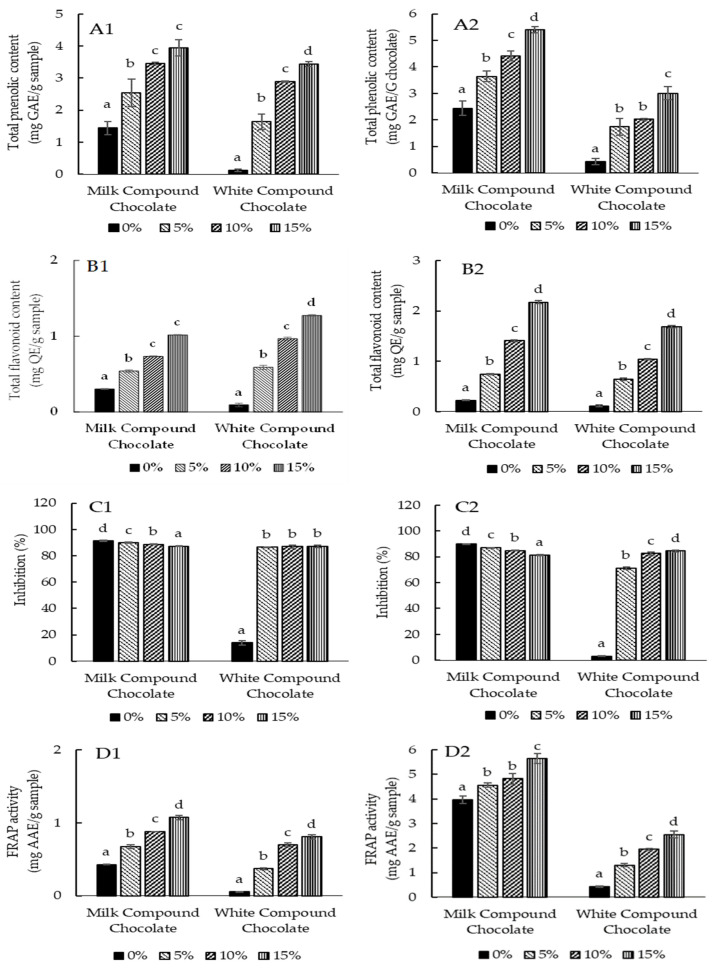
Total phenolic content (**A1**,**A2**), total flavonoid content (**B1**,**B2**), DPPH radical scavenging activity (**C1**,**C2**) and Ferric-reducing antioxidant power (FRAP) activity (**D1**,**D2**) of milk and white compound chocolates formulated with sappan wood (1) and butterfly pea flower (2). The results represented the means of three replicates of independent samples. Mean values within each type of chocolate compound with different lower case letters differ significantly (*p* < 0.05).

As shown in Table 1, sappan wood and butterfly pea flower contained phenols. They exhibited antioxidant activity, and thus, these materials are prospectives for improving the antioxidant properties of milk and white compounds. Figure 1(A1,A2) shows the total phenolic content of milk and white compound chocolates formulated with sappan wood and butterfly pea flower. It was demonstrated that incorporating these additional ingredients significantly improved the total phenolic content of the compounds regardless of the compound type. The percentage of the additional ingredients supplemented in the products was directly proportional to the rise in phenolic content. This is also the case in the total flavonoid content of the formulated chocolate compounds (Figure 1(B1,B2)).

The improvement of the phenolic and flavonoid contents resulted in a significant improvement in the DPPH radical scavenging activity, particularly in the samples of white chocolate compound (Figure 1(C1,C2)). However, incorporating sappan wood and butterfly pea flower could not prevent DPPH radical scavenging activity in the milk chocolate compounds. This discrepancy may be due to the difference in the initial antioxidant activity of the milk and white compounds. Looking back at the DPPH method used in this study, the initial antioxidant content of the milk chocolate compound was likely able to scavenge the DPPH radicals maximally, and thus, the addition of sappan wood and butterfly pea flower could not improve the DPPH radical scavenging activity. In the white chocolate compounds, the initial antioxidant content was low; hence, adding these additional materials demonstrated significant improvement in the DPPH radical scavenging activity. To have a better understanding on the DPPH-radical scavenging activity of the chocolates, the EC_50_ method is recommended for future research. The EC_50_ is defined as the effective concentration required to cause a 50% reduction in DPPH radicals.

Nevertheless, in the FRAP assay, it was confirmed that adding sappan wood and butterfly pea flower significantly improved the antioxidant activity (Figure 1(D1,D2)). As such, the incorporation of 15% powdered sappan wood improved the FRAP activity of the milk chocolate compounds from 0.43 to 1.07 mg AAE/g sample. The improvement was observed in the white chocolate compounds from 0.05 to 0.81 mg AAE/g sample. This result is in excellent agreement with the previous study that worked with chocolate incorporated with cinnamon nanoparticles, emphasizing the strong correlation among concentrated additional materials, total phenolic content and also antioxidant activity [11].

It was found that sappan wood and butterfly pea flower significantly improved the antioxidant activity of milk and white chocolate. The results are in excellent agreement with previous studies conducted by other research groups. As such, Ref. [26] reported that adding Sakura green tea leaves or turmeric powder increased the phenolic concentration of chocolates with 158 distinct phenolic compounds, thus potentially enhancing the health benefits of the formulated chocolates. Raja et al. [33] used *Centella asiatica*, *Abelmoschus esculentus* and *Psidium guajava* in the process of chocolate formulation. The enrichment successfully improved the antioxidant activity of chocolates. Nevertheless, it is also notable that the phenolic profile of enriched chocolates is significantly affected by the novel ingredients introduced in the chocolate formulation. For instance, the incorporation of freeze-dried blueberries, raspberries, blackberries, pomegranates pomace and beet root powders resulted in different phenolic profiles of the innovative chocolates [34].

Theoretically, sappan wood contains brazilin, brazilein, sappan chalcone and protosappanin, while butterfly pea flower contains anthocyanins and various flavanol glycosides of kaempferol, rutin, quercetin and myricetin [14,17]. Therefore, the formulated chocolates are hypothesised to contain those mentioned bioactive compounds. Further research must be undertaken to examine the bioactive compounds profile of the formulated chocolates. It is essential to note that the information regarding these products’ phenolic content and antioxidant activity can only be used for a preliminary prediction. The information is still insufficient to define the overall health effects of the products. Therefore, further study is required to evaluate the bioaccessibility and bioavailability of the bioactive compounds of sappan wood and butterfly pea flower incorporated in the milk and white compound systems.

### 3.3. Colours of Chocolates Formulated with Sappan Wood and Butterfly Pea Flower

Appearance, including colour, is a critical quality attribute of chocolate, since it is the first sensory parameter perceived by consumers prior to the other parameters. Thus, it strongly influences the product acceptability [35]. Table 2 illustrates the effect of the incorporation of powdered sappan wood and butterfly pea flower on the milk and white chocolate compounds. It was shown that both additional materials caused a decrease in chroma values and whiteness index regardless of the type of chocolate compounds. These colour parameters were calculated from the L*, a and b values obtained by a chromameter (L*, a and b values are shown in the Supplementary File). The colour changes were positively affected by the concentration of sappan wood and butterfly pea flower in the formula.

As such the chroma value of the milk chocolate control was 15.7, and the chroma values of milk chocolate formulated with sappan wood 15% and butterfly pea flower 15% were 11.2 and 4, respectively. Meanwhile, the chroma value of the white chocolate control was 20.4, and the chroma values of milk chocolate formulated with 15% sappan wood and 15% butterfly pea flower were 16.3 and 3.6, respectively. Chroma indicates the absolute intensity of an object’s colour. This high disparity in the chroma value designates the colour changes in the samples. This result is also supported by the data of °Hue (the common distinction between colours positioned around a colour wheel) and the whiteness index (the degree of white colour) that decrease significantly after the addition of sappan wood or butterfly pea flower.

**Figure 2:**
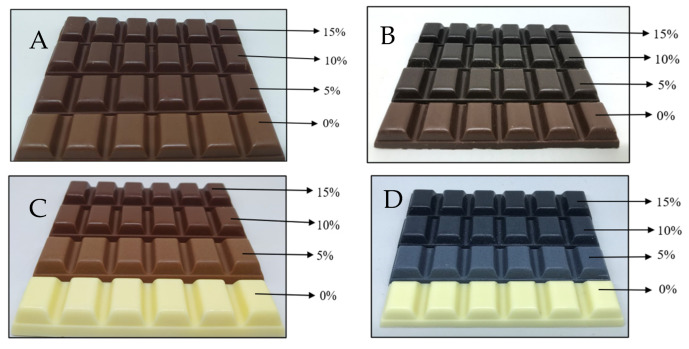
Milk compound chocolates formulated with sappan wood (**A**) and butterfly pea flower (**B**), and white compound chocolates formulated with sappan wood (**C**) and butterfly pea flower (**D**).

It was noticeable that the difference between the formulated white chocolate compound was more pronounced than that of the milk chocolate compound, as indicated in the value of ΔE. This may be because the colour of the additional ingredients is relatively closer to the initial colour of the milk chocolate compounds than that of the white chocolate compounds. However, it is remarkable that the ∆E of all the formulated samples are higher than 3, indicating that the colour differences are apparent to the human eye [36]. Interestingly, adding powdered butterfly pea flower to the white chocolate compound resulted in a blue colour in which the intensity was directly proportional to the concentration of the powdered flower (Figure 2). This result may be an exciting opportunity for the chocolate industry to create a new variant of chocolate products. Adding powdered butterfly pea flower or sappan wood creates darker milk chocolate. Also, it is interesting to note that adding sappan wood powder into white chocolate creates a product with an identical appearance to milk chocolate.

In sum, in addition to the antioxidant properties, enriching compound chocolates with powdered sappan wood and butterfly pea flower improved the colour of the products. The alteration of colour properties in newly formulated chocolate added with functional ingredients has been reported in many studies [37,38,39]. Changing the brightness and whiteness index strongly depends on the type of chocolate and the new ingredients. The different colours of the products formulated with sappan wood and butterfly pea flower were attributed to the natural compounds contained in these materials. As aforementioned, sappan wood is rich in brazilin, while butterfly pea flower is rich in anthocyanins [14,17].

### 3.4. Moisture Content of Chocolates Formulated with Sappan Wood and Butterfly Pea Flower

**Figure 3:**
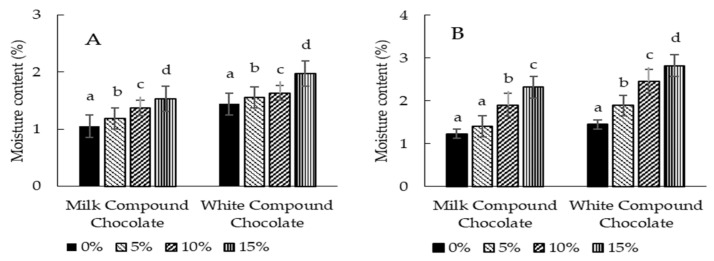
Moisture content of milk and white compound chocolates formulated with sappan wood (**A**) and butterfly pea flower (**B**). The results represented the means of three replicates of independent samples. Mean values within each type of chocolate compound with the different lower case letters differ significantly (*p* < 0.05).

Figure 3 shows the moisture content of milk and white compound chocolates formulated with sappan wood and butterfly pea flower.

The results show that the supplementation of sappan wood and butterfly pea flower significantly increased the moisture content of milk and white compounds. This is reasonable, because the moisture content of the additional ingredients was relatively high. Milk and white compounds contained moisture below 2%, while sappan wood and butterfly pea flower contained moisture of 8.8% and 14.9%, respectively. Therefore, adding the additional ingredients would induce a rise in the moisture content of the milk and white compounds. Chocolate with a lower moisture content (<1%) is highly recommended to delay the physical deterioration due to sugar bloom. However, in many countries, such as in Indonesia (SNI 7934: 2014) [40], the moisture content has not been listed as a quality parameter of chocolate up to now. Much of the literature has shown that the rise in moisture content can substantively influence chocolate’s rheological properties. Shortly, moisture can create a sugar network in milk and white chocolate compounds, strengthening the aggregated particle-to-particle network. In this case, therefore, the product tends to have a higher breaking resistance and be hard to pour [11]. This may also be the case in milk and white compounds. Therefore, an analysis of the apparent viscosity of the milk and white compounds was then carried out in this study.

### 3.5. Flow Properties of Chocolates Formulated with Sappan Wood and Butterfly Pea Flower

The knowledge of chocolate’s rheological behaviour is vital in terms of the processing (pumping and moulding) and sensory properties (mouthfeel). Viscous chocolate tends to have sticky characteristics in the mouth [11]. Table 3 shows the effect of powdered sappan wood and butterfly pea flower on the shear stress and apparent viscosity. As shown, adding these materials significantly increased the above-mentioned rheological parameters. For example, the shear stress of milk compound chocolate increased from 492 Pa to 1347 Pa after being supplemented with 15% powdered sappan wood. It increased to 1232 Pa after being added with 15% of powdered butterfly pea flower. The incorporation also substantially impacts the rise in apparent viscosity from 9853 to 27,484 mPa·s and 24,646 mPa·s, respectively. A similar phenomenon was found in the case of white chocolate compounds. The higher concentration of the additional ingredients added into the formula resulted in a higher shear stress and apparent viscosity.

In this research, the viscosity of the formulated chocolate was affected by the concentrations of powdered sappan wood and butterfly pea flower, in which the viscosity increases with the increasing concentration. The moisture content is the main contributing factor in the change of the rheological properties [11]. The thickening effect in this study may be due to some reasons. The presence of powdered sappan wood and butterfly pea flower increased the moisture content of the milk and white compound chocolates, triggering the formation of a sugar network and resulting in a higher viscosity. Moisture attached to the sugar forms gritty lumps, resulting in higher shear stress, apparent viscosity and torque. In the case of intense gritty lump formation in the chocolate system, more fat coats the lump. Therefore, the amount of “free” fat is reduced; thus, the chocolate has sluggish movement, as the presence of “free” fat is important for a swift flow. The higher concentration of these materials resulted in a more pronounced alteration of the viscosity. Similar findings were found in the previous studies by different research groups. The higher concentration of palm sap sugar with a higher moisture content resulted in a more viscous chocolate [41]. A similar result was also found by Goktas et al. [42], working with paprika extract. Meanwhile, the incorporation of encapsulated green tea extract has been reported to increase the viscosity of white chocolate [43]. Also, the addition of a concentrated extract of raspberry leaf to chocolate resulted in a higher viscosity [44].

Despite the viscosity alteration, the formulation of chocolates and compounds using sappan wood and butterfly pea flower up to 15% can be applied at the industrial level, as it can comply with regulations. As such, Codex Alimentarius (CODEX STAN 87-1981) mentions that the standards of white and milk chocolates focus on the cocoa butter, total milk solids, milk fat, cocoa solids, fat-free solids, non-fat milk solids and milk fat content [45]. According to European Parliament and the Council (Directive 2000/36/EC) [46], the regulation of white and milk chocolates focuses on the content of cocoa butter, dry milk solids, milk fat, cocoa solids, non-fat cocoa solids and total fat (cocoa butter and milk fat). The Food and Drug Administration (Code of Federal Regulations Title 21 Part 163) [47] regulates white chocolate to contain certain levels of cocoa butter, milk fat, total milk solids, nutritive carbohydrate sweetener and emulsifying agents (if applied). Meanwhile, milk chocolate must contain certain levels of cocoa liquor in addition to the afore-mentioned components. Hence, additional ingredients can be added into chocolate or compounds as long as the basic standards of the chocolate or compounds are met.

Thus, this research provides a new strategy and novel product synergizing bioactive compound-rich plants and chocolate, mainly powdered sappan wood and butterfly pea flower. As the products from this study still comply with the standards suggested by food legislation, this product can potentially be commercially produced on an industrial scale. Nevertheless, the limitation of this research lies in the need for more information on particle size distribution. Adding plant-based powders may change the particle size distribution of enriched chocolates, as shown in the previous study [42] working with blackberry capsule powder in white chocolate. However, adding sappan wood and butterfly pea flowers still has mouthfeel properties, as shown in our previous studies [23,24]. It is well known that avoiding gritty texture can be achieved by reducing the particle size to below 30 µm. Thus, to modify the mouthfeel of the enriched chocolate, in terms of grittiness level, an improvement in the grinding duration using the melanger can be carried out, mainly to make smaller particles [48].

## 4. Conclusions

To sum up, it was found that powdered sappan wood and butterfly pea flower contained phenols and flavonoids, and thus, the supplementation of these materials significantly improved the total phenolic and flavonoid contents, as well as the antioxidant activity of milk and white compounds. Supplementation, however, somewhat influences the characteristics of the milk and white compounds. As such, it caused a statistically significant increase in the moisture content of the milk and white compounds due to a high initial moisture content of the raw materials. The supplementation also substantially alters the appearance of the products, resulting in significant lower lightness, chroma values and whiteness index. The degree of difference between the milk and white compounds containing additional ingredients and the control was directly proportional to the concentrations of these materials. Powdered sappan wood and butterfly pea flower caused a statistically significant higher viscosity regardless of compound type. Thus, this study serves as a model for using powdered sappan wood and butterfly pea flower to improve the antioxidant properties of milk and white compounds. Monitoring the stability of the chocolates during storage is important in the future for industrial application. This includes the change in physicochemical properties and the sensory profile of the products, including aroma, sweet taste, acid taste, bitter taste, fracturability, chewiness and elasticity. Also, further studies need to be undertaken to evaluate the bioactive compounds profiles, as well as bioaccessibility and bioavailability of the milk and white compound chocolates enriched with sappan wood and butterfly pea flower.

## Figures and Tables

**Table 1:** Moisture content and antioxidant properties of sappan wood and butterfly pea flower.

Parameter	Sappan Wood	Butterfly Pea Flower
Moisture content (%)	8.8 ± 0.2	14.9 ± 0.2
Total phenolic content (mg GAE/g)	82.4 ± 4.6	19.6 ± 0.5
Total flavonoid content (mg QE/g)	12.6 ± 0.3	9.3 ± 0.2
DPPH (% inhibition)	83.6 ± 0.5	87.7 ± 0.0
FRAP (mg AAE/g)	23.1 ± 1.1	8.3 ± 0.4

Note. The results represent the means of three replicates of independent samples. GAE = Gallic Acid Equivalent; QE = Quercetin Equivalent; DPPH = 2,2-diphenyl-1-picrylhydrazyl; FRAP = Ferric-Reducing Antioxidant Power.

**Table 2:** Colour properties of milk and white compound chocolates formulated with sappan wood (SW) and butterfly pea flower (BF).

Type	Chroma	Hue	Whiteness Index	∆E
**Milk chocolate compounds**				
Choc control	15.7 ± 0.1 ^g^	57.9 ± 0.2 ^c^	34.1 ± 0.2 ^e^	-
Choc SW 5%	13.7 ± 0.1 ^f^	55.5 ± 0.2 ^b^	30.8 ± 0.3 ^d^	4.4 ± 0.5 ^a^
Choc SW 10%	12.3 ± 0.2 ^e^	54.3 ± 0.7 ^ab^	29.9 ± 0.3 ^c^	6.1 ± 0.5 ^b^
Choc SW 15%	11.2 ± 0.3 ^d^	55,0 ± 0.4 ^a^	28.6 ± 0.1 ^b^	8.0 ± 0.2 ^c^
Choc BF 5%	8.0 ± 0.1 ^c^	58.6 ± 0.4 ^c^	30.0 ± 0.3 ^c^	9.6 ± 0.5 ^d^
Choc BF 10%	5.7 ± 0.1 ^b^	59.8 ± 0.5 ^d^	28.3 ± 0.1 ^b^	12.5 ± 0.2 ^e^
Choc BF 15%	4.0 ± 0.1 ^a^	57.9 ± 1.5 ^c^	27.1 ± 0.1 ^a^	14.7 ± 0.3^f^
**White chocolate compounds**				
Choc control	20.4 ± 0.3 ^f^	−73.3 ± 0.2 ^b^	75.6 ± 0.2 ^f^	-
Choc SW 5%	21.9 ± 0.3 ^g^	61.5 ± 0.2 ^g^	40.4 ± 0.3 ^e^	45.1 ± 0.3 ^a^
Choc SW 10%	19.3 ± 0.4 ^e^	58.2 ± 0.5 ^f^	33.9 ± 0.6 ^c^	52.5 ± 0.4 ^b^
Choc SW 15%	16.3 ± 0.1 ^d^	55.7 ± 0.3 ^e^	31.0 ± 0.2 ^b^	56.0 ± 0.3 ^c^
Choc BF 5%	6.3 ± 0.1 ^c^	−75.9 ± 0.6 ^a^	38.7 ± 0.6 ^d^	54.6 ± 0.6 ^d^
Choc BF 10%	5.6 ± 0.2 ^b^	−67.9 ± 1.2 ^c^	31.5 ± 0.7 ^b^	60.8 ± 0.5 ^e^
Choc BF 15%	3.6 ± 0.1 ^a^	−64.0 ± 1.5 ^d^	29.7 ± 0.4 ^a^	61.7 ± 0.1 ^f^

Note: The results represented the means of three replicates of independent samples. The statistical analysis was performed for each type of chocolate. Different superscripts in the same column indicate significant differences (*p* < 0.05) among the samples.

**Table 3:** Flow behaviour of milk and white compound chocolates formulated with sappan wood (SW) and butterfly pea flower (BF).

Type	Shear Stress (Pa)	Apparent Viscosity (mPa·s)	Torque (µN·m)
**Milk Compound Chocolate**			
Choc control	490 ± 20 ^a^	9850 ± 360 ^a^	26,180 ± 970 ^a^
Choc SW 5%	890 ± 80 ^c^	17,760 ± 1670 ^c^	47,200 ± 4440 ^c^
Choc SW 10%	1050 ± 25 ^d^	21,000 ± 500 ^d^	55,820 ± 1340 ^d^
Choc SW 15%	1350 ± 10 ^f^	27,500 ± 170 ^f^	73,026 ± 462.4 ^f^
Choc BF 5%	650 ± 40 ^b^	13,370 ± 240 ^b^	35,520 ± 640 ^b^
Choc BF 10%	850 ± 50 ^c^	16,960 ± 1070 ^c^	45,060 ± 2840 ^c^
Choc BF 15%	1230 ± 20 ^e^	24,640 ± 430 ^e^	65,480 ± 1140 ^e^
**White Compound Chocolate**			
Choc control	150 ± 10 ^a^	3000 ± 190 ^a^	7970 ± 520 ^a^
Choc SW 5%	360 ± 40 ^ab^	7230 ± 780 ^ab^	19,220 ± 2070 ^ab^
Choc SW 10%	400 ± 50 ^bc^	8170 ± 3090 ^bc^	21,720 ± 8220 ^bc^
Choc SW 15%	580 ± 150 ^c^	11,670 ± 3040 ^c^	30,980 ± 8090 ^c^
Choc BF 5%	170 ± 30 ^a^	3420 ± 580 ^a^	9080 ± 1540 ^a^
Choc BF 10%	240 ± 5 ^ab^	4860 ± 110 ^ab^	12,920 ± 280 ^ab^
Choc BF 15%	340 ± 7 ^ab^	6770 ± 140 ^ab^	17,990 ± 370 ^ab^

Note: The results represented the means of three replicates of independent samples. The statistical analysis was performed for each type of chocolate. Different superscripts in the same column indicate significant differences (*p* < 0.05) among the samples.

## Data Availability

The original contributions presented in this study are included in the article/Supplementary Materials. Further inquiries can be directed to the corresponding author.

## Supplementary Materials

The following supporting information can be downloaded at https://www.mdpi.com/article/10.3390/foods13223694/s1: Table S1: The L*, a* and b* values of milk and white compound chocolate formulated with sappan wood (SW) and butterfly pea flower (BF).
