# Supplementary material for: Antioxidant, Physicochemical and Rheological Properties of White and Milk Chocolate Compounds Supplemented with Plant-Based Functional Ingredients
Source: Foods. 2024 Nov 20;13(22):3694. doi: 10.3390/foods13223694 (PMC11594057; doi:10.3390/foods13223694)
Supplement: Supplementary file 1 [file foods-13-03694-s001.zip › foods-3297257-supplementary.pdf]

Supplementary file

Antioxidant, Physicochemical and Rheological Properties of White and Milk Chocolate Compounds Supplemented with Plant-based Functional Ingredients

Elinda Okstaviyani [1+], Puput Dwi Lestari [1+], Kawiji Kawiji [1], Raden Baskara Katri Anandito[2], Anastriyani Yulviatun [1,3], Ardiba Rakhmi Sefrienda[4], and Dimas Rahadian Aji Muhammad [1,3,*]

Table S1: The L*, a* and b* values of milk and white compound chocolate formulated with sappan wood (SW) and butterfly pea flower (BF)

Type	L*	a*	b*
**White Chocolate Compound**			
Choc control	86.62 ± 0.25^{d}	-5.87 ± 0.06^{a}	19.51 ± 0.33^{c}
Choc SW 5%	44.54 ± 0.19^{c}	10.43 ± 0.08^{d}	19.22 ± 0.29^{c}
Choc SW 10%	36.75 ± 0.69^{b}	10.16 ± 0.06^{c}	16.39 ± 0.41^{b}
Choc SW 15%	33.00 ± 0.21^{a}	9.20 ± 0.05^{b}	13.48 ± 0.10^{s}
Choc BF 5%	39.02 ± 0.57^{c}	1.55 ± 0.05^{b}	-6.14 ± 0.10^{a}
Choc BF 10%	31.71 ± 0.67^{b}	2.12 ± 0.18^{c}	-5.23 ± 0.14^{b}
Choc BF 15%	29.82 ± 0.37^{a}	1.60 ± 0.14^{b}	-3.26 ± 0.08^{c}
**Milk Chocolate Compound**			
Choc control	36.04 ± 0.27^{d}	8.37 ± 0.08^{d}	13.32 ± 0.14^{d}
Choc SW 5%	32.16 ± 0.28^{c}	7.75 ± 0.03^{c}	11.26 ± 0.12^{c}
Choc SW 10%	31.02 ± 0.32^{b}	7.20 ± 0.05^{b}	10.01 ± 0.22^{b}
Choc SW 15%	29.50 ± 0.08^{a}	6.61 ± 0.10^{a}	9.09 ± 0.26^{a}
Choc BF 5%	30.42 ± 0.29^{c}	4.15 ± 0.01^{c}	6.79 ± 0.13^{c}
Choc BF 10%	28.55 ± 0.08^{b}	2.86 ± 0.03^{b}	4.92 ± 0.07^{b}
Choc BF 15%	27.16 ± 0.15^{a}	2.14 ± 0.07^{a}	3.40 ± 0.10^{a}

Note: The results represented the means of three replicates of independent samples. The statistical analysis was performed for each type of chocolate. Different superscripts in the same column indicate significant differences ($p < 0.05$) among the samples.
